# Effect of Occupational Cadmium Exposure on Parathyroid Gland

**DOI:** 10.3889/oamjms.2016.042

**Published:** 2016-03-21

**Authors:** Khadiga S. Ibrahim, Safia Beshir, Eman M. Shahy, Weam Shaheen

**Affiliations:** Environmental and Occupational Medicine Department, National Research Centre, Dokki – Giza – Egypt

**Keywords:** cadmium, parathyroid hormone, calcium, magnesium, phosphorus, workers

## Abstract

**BACKGROUND:**

Cadmium (Cd) is used in many industries. High-level exposure is associated with severe kidney and bone damage.

**AIM:**

This study investigates the possible effect of occupational cadmium exposure on parathyroid gland and some minerals in workers.

**METHODS:**

Environmental air monitoring of cadmium was done. Serum and urine cadmium levels, kidney function, some minerals, and plasma parathormone were estimated in the studied groups.

**RESULTS:**

The exposed workers had significantly higher Cd concentration in serum and urine than controls. The mean levels of plasma parathyroid hormone, serum phosphorus and magnesium were significantly lower among the exposed group. However, the mean levels of serum creatinine and calcium were significantly higher in the same group when compared to referents. There was a significant positive correlation between Cd concentration in the serum and urine for the exposed group. The biological Cd exposure indices correlated positively with serum calcium and negatively with plasma PTH level. The prevalence of musculoskeletal complaints, bone ache, joint pain and muscle spasm were more prevalent among the exposed workers compared with the controls with odds ratio 4.316, 3.053 and 3.103 respectively.

**CONCLUSIONS:**

Occupational cadmium exposure has an adverse effect on PTH level and serum human minerals.

## Introduction

Cadmium (Cd) is a heavy metal used in the fabrication of batteries, as a color pigment, and as an anticorrosive agent. Normal people can be exposed to Cd through contaminated air, food, and water. Also, smoking is another environmental source of Cd [1].

Cadmium resorption occurs through gastrointestinal tract, pulmonary system and skin. The kidney is the main organ for long-term cadmium accumulation.

Liver and kidney can change cadmium to a harmless form but too much cadmium can overload their ability to change it [2].

Bone remodeling cycle is a normal process by which bone is continually repaired and renewed. Osteoporosis develops when there is an imbalance between bone formation and bone resorption. Osteoporosis is characterized by low bone mass with an increase in bone fragility and susceptibility to fracture [3].

Several studies found that exposure to low concentrations of Cd affects bone mineral density (BMD) which may increase the risk to osteoporosis and fractures [4]. Exposure to cadmium may reduce the amount of active form of vitamin D (1, 25(OH)2D) produced in the kidney. This delays calcium uptake in the duodenum and active calcium reabsorption in the distal convoluted tubule which is the main mechanism of osteoporosis [5]. Moreover, Cd may affect directly osteoblast and osteoclast functions diminishing their ability to mineralize and collagen production [6].

Chronic exposure to Cd affects the structure and function of the parathyroids in male rats. The effects occur at low Cd accumulation and potentiate with the intensity and duration of exposure and body burden of Cd [7]. Cd occupational exposure has for long been associated with hypercalciuria, renal tubular cell dysfunction and osteoporosis [3].

Bone effects of occupational Cd exposure in apparently healthy men is unclear, therefore, this study was performed to investigate the possible effect of occupational cadmium exposure on parathyroid gland and some minerals among a group of workers occupationally exposed to Cd which may give an idea about the changes associated with such exposure.

## Materials and Methods

### 

#### Subjects

This is a cross-sectional comparative study that included 51 electroplating male non-smoker workers exposed to cadmium from a factory in the industrial city El-Sadat, Menofia governate in Egypt with a mean age 37.6 ± 9.2 yr. and duration of exposure ranged between 10-28 yr (15.95 ± 6.9). A control group of 43 male nonsmoker employees matched for age (36.9 ± 7.5 yr) and social class with no past or present history of occupational exposure to Cd was included. Subjects having renal problems, diabetics, using calcium supplements or vitamin D therapy were excluded from this work. The subjects investigated did not use any personal protective equipment.

From all participants, a written consent was obtained after the nature of the study has been explained in full detail. Also, they were interviewed and completed a questionnaire that included personal data, detailed occupational history (past and present), musculoskeletal complaints and medical history.

#### Sampling

Venous blood samples were collected with 5 ml syringes and divided into 2 parts, one part collected as clotted blood and centrifuged to separate serum to estimate urea, creatinine (Cr), albumin, calcium, phosphorus, alkaline phosphatase (ALP), and magnesium (Mg). The other part of blood sample was collected on an anticoagulant EDTA and centrifuged to separate plasma to estimate PTH. The serum and plasma samples were immediately frozen at –20°C until analyzed. Urine specimens were collected in plastic containers previously acid washed by 1M HNO_3_. Also, the urine was frozen and kept at –20°C until analyzed.

#### Monitoring

a) Environmental monitoring: at selected workplaces, dust measurements were carried out by air sampling from breathing zones. Determination of Cd in dust samples was carried out by graphite furnace atomic absorption spectrometry.
b) Biological monitoring: for both exposed and referent groups:
  Urine samples were analyzed for Cd and Cr. Cadmium concentration in urine was determined by graphite furnace atomic absorption spectrometry [8]. The standard addition technique was used for calibration. The cadmium content of urine was expressed as micrograms per gram Cr (μg/g Cr) excretion to compensate for an effect of muscle mass on Cr excretion.

- Serum Cd concentration was determined by graphite furnace atomic absorption spectrometry [8].

- Serum urea was determined by urease modified Berthelot reaction [9].

- Serum and urine creatinine were determined by Jaffe method without deproteinization [10].

- Serum albumin was determined according to Doumans et al [11].

- Serum calcium was determined by spectrophotometric method [12].

- Serum phosphorus was determined according to Goodmin [13].

- Serum alkaline phosphatase was determined according to Belfield and Goldberg [14].

- Serum magnesium was determined according to Vassault et al [15].

- Plasma PTH was determined by ELISA according to Endres et al [16].

#### Statistical analysis

The data was statistically analyzed using the SPSS18 program. Independent t-test and Chi-square test were used to detect the statistical differences in the quantitative and qualitative data respectively between the two groups. Pearson’s bivariate correlation coefficient was also calculated. The differences were considered significant at a level of p < 0.05.

## Results

The time-weighted average concentration of Cd in the air was estimated during normal work. The air cadmium level ranged from 21 to 26 μg/m^3^ with a mean level of 23 ± 0.002 μg/m^3^.

There was no significant difference between the exposed and the control groups as regards their age (p>0.05).

Mean levels of Cd in both serum and urine were much higher in the exposed group compared to the controls and the differences were highly statistically significant (p < 0.01) as shown in Table 1.

**Table 1:** Blood and urinary cadmium concentrations in the exposed and referents groups

Parameters	Exposed (51)	Control(43)	P-value
Serum Cd (μg/dL)	3.09 ± 1.8	0.84 ± 0.19	<0.001
Urine Cd (μg/g Cr)	9.36 ± 2.43	1.98 ± 0.54	<0.001

It was observed from Table 2 that the mean levels of serum Cr and calcium were significantly higher in the exposed workers than in the controls. While, the mean values of plasma PTH, serum phosphorus, and Mg were significantly lower in the exposed group than in the referent group. There was no significant difference between both groups as regards serum urea, albumin, and ALP.

**Table 2:** Different biochemical variables of studied groups

Parameters	Normal range	Exposed (51)	Control (43)	P-value
Serum urea (mg/dl)	Oct-50	34.1 ± 9.6	32.1 ± 5.66	>0.05
Serum Cr (mg/dl)	0.8-1.2	1.10 ± 0.16	0.82 ± 0.11	<0.001
Serum albumin (gm/dl)	3.5-5.2	4.11 ± 0.3	4.3 ± 0.5	>0.05
Plasma PTH (pg/ml)	Oct-65	33.1 ± 7.1	51.1 ± 10.4	<0.001
Serum calcium (mg/dl)	8.5-10.5	10.24 ± 1.48	9.34 ± 0.71	<0.001
Serum phosphrous (mg/dl)	2.5 - 4.5	2.11 ± 0.74	3.61 ± 0.79	<0.001
Serum ALP (U/dl)	13-Mar	9.72 ± 1.67	12.03 ± 1.86	>0.05
Serum Mg (mg/dl)	1.8 - 2.6	1.56 ± 0.37	2.30 ± 0.29	<0.001

The prevalence of musculoskeletal complaints (Table 3), bone ache, joint pain and muscle spasm were more prevalent among the exposed workers compared with the controls (p < 0.05) with odds ratio 4.316, 3.053 and 3.103 respectively.

**Table 3:** Musculoskeletal complaints among the studied groups

Complaints	Exposed (51)		Control(43)		χ^2^	95% CI		Odds Ratio
	No	%	No	%				
						Lower limit	Upper limit	
Bone ache	21	37.7	6	17.0	<0.007	1.545	12.057	4.316
Joint pain	19	34.0	7	14.6	<0.041	1.136	8.208	3.053
Muscle spasm	23	39.6	9	19.5	<0.024	1.238	7.776	3.103
Fractures	3	5.9	2	4.2	>0.050			

The correlation between biological Cd exposure indices (serum and urinary Cd levels) and the other biochemical parameters are shown in Table 4. There was a significant positive correlation between Cd concentration in the serum and urine for the exposed workers (r = 0.362). Also, there was a significant positive correlation between the biological Cd exposure indices and serum calcium (r = 0.230, p < 0.05 with serum Cd, and r = 0.275, p < 0.05 with the urinary Cd). While there was a significant negative correlation between the Cd exposure indices and plasma PTH levels (r = -0.412, p < 0.01 with the serum Cd, and r = -0.478, p < 0.01 with the urinary Cd.

**Table 4:** Correlation between biological Cadmium exposure indices and different studied parameters+

Variable		urea	Cr	Albumin	PTH	calcium	Phosphorous	ALP	Mg	Serum Cd	Urine Cd
SerumCd	r	0.058	0.097	0.088	- 0.412**	0.230*	0.016	0.113	0.019	1.000	0.362**

Urine Cd	r	0.114	0.077	0.091	- 0.478**	0.275*	0.192	0.010	0.189	0.362**	1.000

* Significant at the level of P < 0.05;

** Significant at the level of P < 0.01.

## Discussion

In the current study, workers were exposed to poor workplace environment which is considered about 4 folds compared to Occupational Health and Safety Administration [17] permissible exposure limit (PEL) 5 μg/m^3^. Moreover, the workers in the present study didn’t use personal protective devices provided by the occupational safety department in the factory. This may have contributed to their high serum and urinary Cd levels.

U-Cd level assesses long-term exposure to cadmium because it reflects the cadmium accumulation in the kidney; however current exposure is assessed by Cd level in blood [18]. In our study, as expected, the serum and urinary Cd concentrations of the exposed group were much higher than those of the non-exposed group as shown in Table 1. These results agreed with some previous studies who found significant higher urinary Cd concentrations among the workers compared to the controls [19-21]. Also, our results were in accordance with another study who found higher serum Cd concentration among the workers exposed to Cd compared to controls [22]. Other investigators [19, 23] found a linear correlation between Cd concentration in serum and urine. The data is consistent with our finding where a significant positive correlation was found between the Cd concentrations in the serum and urine for the exposed group.

The current study results revealed absence of renal dysfunction of the exposed group as serum levels of urea and creatinine are within the normal range on one side, which agreed with previous studies results [19, 22], on another side absence of renal dysfunction was confirmed by normal alkaline phosphatase level which was in accordance with another earlier study [24].

Cadmium is suggested to have a direct osteotoxic effect even in the absence of renal dysfunction as occupational exposure to cadmium is associated with lower body mineral density and a higher urinary calcium excretion [25].

Alkaline phosphatase estimation assesses bone turnover in chronic kidney disease patients, especially with elevated PTH levels [24]. The present study showed normal alkaline phosphatase level in the exposed group suggesting the absence of renal or bone affection.

In this study, there was a significant decrease in the mean values of plasma PTH, serum phosphorus, and magnesium while there was a significant increase in the mean value of serum calcium in the exposed group compared with the controls.

PTH is the most important endocrine regulator of calcium and phosphorus concentration in the extracellular fluid. Once in the circulation, PTH binds to PTH receptors that are located throughout the body. Thus, disorders of PTH excess or insufficiency not only affect serum levels of calcium and phosphorus but also affect bone, cardiac, skin, neurologic and other systemic manifestations. PTH secretion occurs in response to hypocalcemia and hyperphosphatemia and is inhibited by severe hypomagnesemia [26].

A study done on male rats indicated that the critical Cd concentration for the parathyroids may be lower than that for the kidney. Damage to the parathyroids at low Cd concentration suggests that the damaging effect of Cd may be caused by its indirect rather than direct action [7].

Cadmium increased levels were associated with increased bone resorption and lower secretion of PTH, but not with markers of bone formation [4]. Thus, the lower levels of PTH are most likely a compensatory decrease in the increased skeletal release of calcium due to enhanced bone resorption. Phosphorus is important for the modulation of calcium mobilization from the bone and the regulation of plasma calcium. Increase in serum calcium is associated with a decrease in serum phosphorus and increased urinary phosphorus excretion and vice versa [27].

Hypoalbuminemia is avoided in the current study as in the presence of hypoalbuminemia, total serum calcium may be underestimated [28].

In all patients with hypercalcemia, the PTH level should be suppressed. Thus, interpretation of a PTH level must always be done in conjunction with a simultaneous calcium level. If a serum calcium level is 11.5 mg/dl and the PTH is 50 pg/ml then there is an inappropriate circulating level of PTH since it should be suppressed. Thus, despite the PTH is in the normal range (usually 10–65 pg/ml), it is indicative of hyperparathyroidism. However, if the calcium level was 8.0 mg/dl this level of PTH would be normal [29], this might explain the hypercalcemia and low normal phosphorus found in the exposed group despite their apparently normal PTH serum level.

Although age, blood urea, serum albumin, and the nutritional state may influence the PTH level [30], lack of any significant change in the present study suggests that probably they do not contribute to the Cd-induced parathyroid effects.

In the present study exposed workers showed lower Mg level compared to the controls, which agreed with a previous study who investigated occupational exposure to cadmium and its effect on elements in Nickel - Cadmium battery production workers [20]. Low blood Mg levels, thought to be a result of Cd exposure, could be explained by an increased Cd level in the gut. It is induced by mucociliary transfer by bile elimination or by excretion of Cd through the intestine walls which in turn may cause an inhibition of Mg absorption in gut thus leading to a decrease of blood Mg [31]. A recent study concluded that Cd uptake in human osteoblastic cells occurs, at least in part, through Ca- and Mg-inhibitable transport mechanisms [32].

As regards the prevalence of musculoskeletal complaints (Table 3), bone ache, joint pain and muscle strain were more prevalent among the exposed workers compared to the controls. This may be referred to hypercalcemia, hypomagnesemia and hypophosphatemia found in the exposed group.

The negative correlation between the Cd exposure indices and plasma PTH levels confirm the relationship between the damaging Cd impact on the parathyroids and the intensity of exposure and body burden of Cd as confirmed by a previous study [7].

In conclusion, it is apparent from our study that Cd exposure may have an adverse effect on PTH level and serum human minerals. Even in the absence of renal dysfunction, prominent parathormone level together with hypercalcemia as well as low normal phosphorus are basically indicative of primary hyperparathyroidism.

Occupational cadmium exposure health hazards can be lessened through individual caring equipment, fine sanitation practices, manage and diminution of cadmium emissions. Alternatively, Mg supplementation [33] has a useful function in lowering Cd body load and might be intended for preclusion and/or treatment. Further studies on a larger scale are recommended.
